# Imipenem/cilastatin encapsulated polymeric nanoparticles for destroying carbapenem-resistant bacterial isolates

**DOI:** 10.1186/s12951-017-0262-9

**Published:** 2017-04-11

**Authors:** Mona I. Shaaban, Mohamed A. Shaker, Fatma M. Mady

**Affiliations:** 1 Pharmaceutics and Pharmaceutical Technology Department, College of Pharmacy, Taibah University, PO Box 30040, Al Madina, Al Munawara Saudi Arabia; 2 Microbiology and Immunology Department, Faculty of Pharmacy, Mansoura University, PO Box 35516, Mansoura, Egypt; 3 Pharmaceutics Department, Faculty of Pharmacy, Helwan University, PO Box 11795, Cairo, Egypt; 4 Pharmaceutics Department, Faculty of Pharmacy, El-Minia University, El-Minia, Egypt

**Keywords:** Imipenem, Antibiotic resistance, Biodegradable, PCL, PLGA, Nanoparticles

## Abstract

**Background:**

Carbapenem-resistance is an extremely growing medical threat in antibacterial therapy as the incurable resistant strains easily develop a multi-resistance action to other potent antimicrobial agents. Nonetheless, the protective delivery of current antibiotics using nano-carriers opens a tremendous approach in the antimicrobial therapy, allowing the nano-formulated antibiotics to beat these health threat pathogens. Herein, we encapsulated imipenem into biodegradable polymeric nanoparticles to destroy the imipenem-resistant bacteria and overcome the microbial adhesion and dissemination. Imipenem loaded poly Ɛ-caprolactone (PCL) and polylactide-*co*-glycolide (PLGA) nanocapsules were formulated using double emulsion evaporation method. The obtained nanocapsules were characterized for mean particle diameter, morphology, loading efficiency, and in vitro release. The in vitro antimicrobial and anti adhesion activities were evaluated against selected imipenem-resistant *Klebsiella pneumoniae* and *Pseudomonas aeruginosa* clinical isolates.

**Results:**

The obtained results reveal that imipenem loaded PCL nano-formulation enhances the microbial susceptibility and antimicrobial activity of imipenem. The imipenem loaded PCL nanoparticles caused faster microbial killing within 2–3 h compared to the imipenem loaded PLGA and free drug. Successfully, PCL nanocapsules were able to protect imipenem from enzymatic degradation by resistant isolates and prevent the emergence of the resistant colonies, as it lowered the mutation prevention concentration of free imipenem by twofolds. Moreover, the imipenem loaded PCL eliminated bacterial attachment and the biofilm assembly of *P. aeruginosa* and *K. pneumoniae* planktonic bacteria by 74 and 78.4%, respectively.

**Conclusions:**

These promising results indicate that polymeric nanoparticles recover the efficacy of imipenem and can be considered as a new paradigm shift against multidrug-resistant isolates in treating severe bacterial infections.

## Background

Over the last decades, the frightening spread of antibiotic-resistant infections all over the world inherently emerges multidrug-resistant/pan-resistant pathogens that evade even powerful antibiotics [1]. Various strategies have been used by the bacteria to develop resistance, including secretion of antibiotic specific/nonspecific inactivation enzyme, active efflux of antibiotics and surfaces adhesion with the formation of a protective biofilm [2, 3]. This biofilm provide an inaccessible barrier to even small molecule antibacterial agents affording the suitable support for the bacterial proliferation/colonization and development of a severe health threatening microbial infections [2]. Consequently, searching for effective and biofilm preventing bactericidal agents is deemed necessary in the clinical prospective of antibacterial therapy [4]. Designing new generation or derivative of antibiotics is incredibly costly investment process and it wastes much time until it is distinguished in the pharmaceutical production pipelines, however, protection through a smart delivery system can potentiate the bactericidal efficacy of existing antibiotics and adequately address a solution to cease the current progression of resistant bacteria [5].

Carbapenems are new broad spectrum beta lactam antibacterial agents with potent activity against serious/complicated bacterial infections, which keeps them as the last reliable hospitalization treatment line for the life threatening microbial infections [6]. Initially, they had demonstrated a great stability against the hydrolysis by the beta-lactamases produced from the resistant pathogens, however, the emergence of carbapenem-resistance has been noticed globally with Gram-negative pathogenic bacteria such as *Enterobacteriaceae* and *Pseudomonas*. The prevalence of such resistance has been associated mainly with the bacterial formation of carbapenemases (carbapenem hydrolyzing enzymes), elimination of carbapenem influx, activation of the multi-drug efflux pump and mutation of the outer membrane protein [7, 8]. Such developed resistance rapidly disseminate between isolates and even among various species via integrons, transposons and exchange of plasmids [8]. Hazardously, carbapenem-resistant strains have been recently found to be associated with resistance to an expansive diversity of antimicrobials such as aminoglycosides, quinolones and cephalosporins [1]. Facing the aforementioned therapeutic challenge, various approaches have been investigated to formulate/deliver carbapenems, trying to accomplish two main goals. The first one is to overcome the stability issue through protecting the carbapenems molecular entity from the degradation bacterial enzymes to circumvent this bacterial blow [2]. The second is to target carbapenems to their site of action through increasing their bacterial penetration/uptake to predominate their therapeutic action on the bacterial renitences. The research efforts to reach these two goals are still ongoing.

Nano-size carriers claim the sufficient chemical protection and the adequate targeting effect needed for the effective delivery of antimicrobial molecules [5]. These nano-carriers include but not limited to liposomes, solid lipid nanoparticles, polymeric nanoparticles, metal nanoparticles, quantum dots and self-assembled micelles [9–12]. An extensive evaluation for the pros and cons of each of those nano-carriers for combatting microbial resistance has recently been reviewed [4, 5, 11–14]. Reader is strongly advised to refer/read those reviews for detailed discussions related to the improvement in the antibacterial activity of each of those nano-carrier delivery systems against various pathogenic microbes [11, 12]. Among the previously mentioned nano-size carriers, using polymer nanoparticles for the delivery of antibiotics has been growing steadily over the last years and getting special attention in the landscape of microbial resistance as well as prevention/eradication of biofilm formation [13, 14]. Polymeric nanoparticles are considered a promising antibiotics delivery vehicle due to the appropriate thermodynamic stability of the prepared self-assembled nano-sized particles. In addition to their ability to increase bacterial uptake and the penetration power of the loaded antibiotics to combat the developed multi-drug resistances. Meantime, they provide more in vivo stability for the loaded antibiotics against biodegradation, increase the circulation time inside the body and decrease the therapeutic dose and administration frequency [15]. Also, polymeric nanoparticles enhance localization of the loaded antibiotics to the infected organ, which is associated with minimizing the side effects accompanying the common systemic administration [16].

The highlighted biodegradable polymers that are widely used in literature as pharmaceutical carriers are either natural (such as alginate and chitosan) [17], or synthetic polyester from alpha hydroxy-acids (such as polylactide, polyglycolide and poly Ɛ-caprolactone) [12] and polyamino acids [18]. Meantime, the only reported study for imipenem delivery was by Fazli et al. who physically entrap imipenem/cilastatin in the nanopores of ZnO nanoparticles and incorporate it with chitosan–polyethylene oxide nanofibrous mat [17]. Successfully they were able to sustain the release of loaded imipenem/cilastatin, however, there are still some unresolved issues and problems surrounding the potential toxicity of using ZnO nanoparticles in pharmaceutical applications [19]. On the other hand, polyhydroxy-acid ester are brilliant biodegradable biomaterials that have been employed as a promising vehicle to antimicrobials delivery in a controlled/sustained categorical form for a distinct period of time [20]. The specification of used polymer (e.g. molecular weight, hydrophilicity/hydrophobicity) easily manipulated to tailor the prepared nanoparticles for a specific delivery purpose [20]. To our knowledge, no research study has evaluated the using of polyhydroxy-acid esters for the protective delivery of carbapenems as explored in this study for the first time. Herein, we selected imipenem as the first candidate drug to represent carbapenems and in combination with cilastatin. Cliastatin has no antibacterial activity but inhibits the enzymatic degradation of imipenem molecules occurs by the renal dehydropeptidase [17]. In this paper, we formulated imipenem/cilastatin as polymeric nanocapsules using polylactide-*co*-glycolide (PLGA) and poly Ɛ-caprolactone (PCL). We also characterized the prepared nanoparticles and evaluated their in vitro antibacterial efficiency against selected imipenem-resistant isolates.

## Methods

### Bacterial isolates and growth setting

The bacterial isolates were collected from clinical laboratory, Mansoura University Hospitals, Mansura, Egypt. *Klebsiella pneumoniae* isolates KMU5.5, KMU4.5, KMU2.3 were separated from urine samples. *Pseudomonas aeruginosa* isolates PUMU2.3, PWMU2.3 and PSMU8.0 were obtained from urine, wound and sputum samples, respectively. Isolates were identified according to the standard procedures of Bergey’s manual, 1989 [21]. The sensitivity of the collected isolates to imipenem was first detected through the standard disc diffusion method (CLSI, 2015) [22]. Non-imipenem resistant standard strain *Klebsiella pneumoniae* ATTCC 4352 and *Pseudomonas aeruginosa* PAO1 were used as negative control. All the collected strains were propagated in Luria–Bertani (LB) medium (Yeast extracts 0.5%, Tryptone 1% and Sodium Chloride 1%, pH 6.8).

### Materials

Poly Ɛ-caprolactone (Mn = 45,000), polylactide-*co*-glycolide (50:50 Mw = 7000–17,000) and didodecyl dimethyl ammonium bromide (DMAB) were obtained from Aldrich-Sigma chemical company, USA. Imipenem and cilastatin (pharmaceutical grade) were kindly gifted from Merck Sharp & Dohme Corp., Canada. All the other chemicals were of analytical grade and used as received. Deionised water (Millipore^®^, 18.2 MΩ cm) was utilized as the water source in the experimental procedures.

### Preparation of imipenem/cilastatin encapsulated nanoparticles

Polymeric nanocapsules prepared by double emulsion evaporation technique [23]. Briefly, 20 mg of both imipenem and cilastatin were first dissolved in 4 ml of deionized water. Sixty milligrams of the used polymer (either PCL or PLGA) were dissolved in 8 ml of chloroform. The antibiotic solution was blended with the polymer solution and emulsified by using a probe homogenizer (IKA, Ultra-Turrax) at 20,000 rpm for 15 min, to form the primary w/o emulsion and 0.2 ml of ethyl cellulose solution (10% w/v in chloroform) was added as a stabilizer for the prepared emulsion. Then, 25 ml of 1% w/v DMAB aqueous solution was added directly into the prepared w/o emulsion and emulsified by using a probe homogenizer at 20,000 rpm for 15 min, to produce the multiple-emulsion (w/o/w). The obtained emulsion was sonicated for 2 min and diluted with 20 ml of deionized water. The chloroform was then evaporated under vacuum by stirring for further 2 h at room temperature. The produced nanoparticles were collected by centrifugation for 20 min at 20,000 rpm and then rinsed three times by deionized water. In the following discussion, IMP refers to the physical mixture of equal weight of imipenem and cilastatin, however, IMP/PCL and IMP/PLGA refer to imipenem/cilastatin loaded poly Ɛ-caprolactone and polylactide-*co*-glycolide nanoparticles, respectively.

### Size and morphology of the prepared nanoparticles

Laser diffraction was used for particle analysis through measuring the particle size and zeta potential for the prepared nanoparticles (Microtrac, nanotrac wave II Q). Before measurement, all the samples were properly sonicated to avoid any aggregation and diluted with deionized water.

The shape and topology of the nanoparticles were detected by a Bioscope Catalyst-atomic force microscope (Bruker, Santa Barbra, CA, USA) mounted on Lica inverted microscope (Wetzlar, Germany). A single ScanAsyst-Fluid probe with a tip radius of 20 nm (Bruker, Santa Barbra, CA, USA) was used. The deflection sensitivity (nm/V) was determined on a glass and was found to be 45.5 nm/V. The nanoparticles suspension was placed on the silicon wafer and air dried.

### Measurement of encapsulation efficiency, drug loading and nanoparticle yield

The encapsulation efficiency (EE) and drug loading (DL) were simply measured by quantizing the imipenem and cilastatin encapsulated inside the obtained nanoparticles after being disassembled and dissolved with methanol. The imipenem and cilastatin concentrations were determined by measurement of their UV absorbance at 318 and 243 nm, respectively, using a UV/visible spectroscopy (Evolution 201 UV–visible spectrophotometer, Thermo scientific). Simultaneously, nanoparticles’ yield (NY) was determined gravimetrically. The percentage of the EE, DL and NY were calculated as follows:

$${\text{EE}}\% = \left( {{\text{weight of encapsulated drug in nanoparticles}}/{\text{initial weight of added drug}}} \right) \times 100,$$

$${\text{DL}}\% = \left( {{\text{weight of encapsulated drug in nanoparticles}}/{\text{weight of nanoparticles}}} \right) \times 100,$$

$${\text{NY}}\% = \left( {{\text{weight of nanoparticles}}/{\text{initial weight of added drug and polymer}}} \right) \times 100.$$

### Fourier transform infrared

Fourier transform infrared (FTIR) spectroscopic analysis was performed by using IRAffinity-1S spectrophotometer (Shimadzu, Japan) supplied with sealed interferometer with auto dryer attenuated total reflection accessory, and dynamic alignment system. All spectra were obtained at room temperature with examining spectrum of 400–4000 cm^−1^. Each sample scanned for 32 times through a mercury cadmium telluride detector and the resolution of the spectrum was 4 cm^−1^ for all step-scan FTIR measurements.

### X-ray powder diffraction analysis

X-ray powder diffraction (X-RPD) analysis were obtained using the Bruker D8-Advance X-ray diffractometer (Bruker, Germany). Scanning was implemented at a voltage of 40 kV and 30 mA using Cu Kα radiation. The scanned angle was set from 5° to 80° with accuracy of 0.02 throughout the measurement range, and the scanned rate was 4°/min.

### Antimicrobial effect of the prepared nanoparticles

Primary screening of antimicrobial activity of IMP, IMP/PCL and IMP/PLGA was carried out by agar diffusion assay. The activity of IMP/PCL and IMP/PLGA against imipenem resistant isolates was compared to IMP. The minimal inhibitory concentrations (MICs) of IMP and its nano preparations were quantified throughout the microtiter plate assay method [22]. Muller–Hinton (100 µl) was distributed in a sterile microtitre plate. Twofold serial dilutions of IMP, IMP/PLGA, and IMP/PCL were prepared 1250, 625, 312.5, 156.5, 78.12, 39, 19.5, 9.75, 4.87, 2.4, 1.2 and 0.6 µg/ml in Muller–Hinton broth medium. All dilutions were inoculated with imipenem resistant isolates at final concentrations of 0.5 × 10^5^ CFU/ml. Wells for positive and negative controls were included with all experiments. The MICs of plain nanoparticles of PLGA and PCL were also determined under the same conditions. The plates were incubated at 37 °C for 24 h and the minimum concentration that inhibit the growth of bacteria was determined.

### Mutation prevention concentration (MPC)

The MPC was determined for IMP, IMP/PCL and IMP/PLGA according to the agar plate dilution procedures as reported previously by Credito et al. [24]. Briefly, twofold serial dilutions of the tested preparations (0.5–16 × MIC) were incorporated into Mueller–Hinton agar plates. Each plate was inoculated with 50 µl of a concentrated bacterial suspension containing 10^10^ CFU/ml. The plates were incubated for 24 h at 37 °C and each dilution was tested in triplicate. The isolates growing on the plates supplemented with antibiotic concentration ≥MIC were transferred on antibiotic-free medium, followed by a redetermination of their MICs to assess the development of microbial resistance. MPC was estimated as the concentration of the first plate that showed no bacterial growth.

### Effect of carbapenemase on IMP, IMP/PCL and IMP/PLGA

The ability of the polymeric nano-formulation to protect imipenem from degrading enzymes was studied. *Pseudomonas aeruginosa* and *Klebsiella pneumoniae* isolates were first tested for carbapenemase production by using Modified Hodge Test (MHT) [22]. Carbapenemase enzymes were then isolated from carbapenemases producing strains by applying the published method by Masuda et al. with some modifications [3]. The isolates were propagated by overnight incubation at 37 °C in 40 ml LB medium. Bacterial pellets were collected by centrifugation at 4000 rpm for 10 min. Cells were suspended in equal volume of phosphate buffered saline (PBS) and disrupted by five cycles of sonication (30 s each) on ice. Crude enzymes in the supernatant were separated through centrifugation for 10 min at 8000 rpm. The presence of carbapenemase in the cell extracts was tested microbiologically by monitoring the hydrolysis of imipenem.

Finally, the stability of the nano-preparations was evaluated in the presence/absence of degrading enzymes compared to IMP. In this study, IMP, IMP/PCL and IMP/PLGA (1 mg/ml) were mixed with 100 µl of the enzyme extract and incubated for 1 h at 37 °C. Mueller–Hinton agar plates were inoculated with *Escherichia coli* (ATCC 25922) with adjusted turbidity to 0.5 McFarland standard. Wells were performed in the plates for IMP, IMP/PCL and IMP/PLGA either treated or untreated with the degrading enzyme and each sample was applied to the corresponding wells. The plates were incubated at 37 °C overnight for detection of growth inhibition zone.

### Antibiotic kill test

The rate of killing imipenem-resistant bacteria by IMP/PCL and IMP/PLGA was determined and compared to IMP free antibiotic. KMU5.5 was propagated till bacterial count 5 × 10^6^ CFU/ml. The culture was mixed with 5 µg/ml of each IMP or IMP/PCL or IMP/PLGA. Samples were taken at zero, 1, 2, 3, 4, 6, 10, and 24 h and each sample was serially diluted 1:10 to determine the number of the viable bacteria. In the time kill assay of PUMU2.3, 10 µg/ml of each preparation were used. Bacterial growth without IMP or IMP formulated particles was also tested. The number of bacteria recovered overtime post treatment was determined by surface drop method in triplicates [25]. The viable count (CFU/ml) = dilution factor × (Average number of colonies/drop)/volume of drop (0.01 ml). The count of the recovered cells post antibiotic treatment was plotted as the CFU/ml over time. The experiment was performed in triplicate.

### Effect on bacterial attachment

The efficacy of the formulated nano-imipenem on microbial adhesion and biofilm formation was evaluated using microtitre plate method [26]. Growth medium containing 1/4 MICs of IMP, IMP/PCL and IMP/PLGA were prepared and 100 μl of each preparation was distributed into wells in triplicates. Overnight cultures of the tested isolates were diluted and 10 μl of each bacterial suspension was placed in the corresponding well and the plates were incubated at 37 °C for 24 h. Microbial controls containing no antibiotic were performed under the same conditions. At the second day, the wells were aspirated and free cells were washed with saline and attached biofilms were fixed with methyl alcohol. The wells were stained with crystal violet for 15 min and the plates were washed with deionized water. The stained biofilm with crystal violet was dissolved with glacial acetic acid (33% v/v) and measured with an E max microtitre ELISA reader (Microplate reader, Per long Medical Equipment Co., Ltd. China) at OD 490 nm and the mean reading of three wells was calculated [27].

### In vitro drug release

Membrane dialysis was used for studying the in vitro release of imipenem and cilastatin. Nanoparticles were first suspended at a concentration of 10 mg/ml in PBS pH = 7.4 and then transferred to a pre-soaked Fischer dialysis tube (MWCO = 12,000–14,000 daltons). The assembled dialysis bags were immersed into 100 ml PBS of pH = 7.4 at 37 °C under shaking (100 rpm/min). At different time intervals, 4 ml of the release buffer was withdrawn and returned back after being measured by UV/vis spectrophotometer (Evolution 201 UV–visible spectro-photometer, Thermo scientific) to calculate the released amount of both imipenem and cilastatin.

### Statistical analysis

Statistical analysis of the data was manipulated by using two-way ANOVA followed by the Bonferroni post-tests, using GraphPad Prism version 5.02 (GraphPad Software, Inc. La Jolla, USA). Data represented as mean ± standard deviation (SD). Statistical differences between the groups were considered significant if the *p* value was <0.05.

## Results and discussion

Imipenem-resistant pathogens account for serious public and hospital-gained infections [28]. Restoring the antibacterial properties of imipenem modifies this health care concern and polymeric nano-encapsulation shall be the approach to catch that desire. Herein, we prepared polymeric nanocapsules by double emulsion formation followed by organic solvent evaporation [23]. The principles to generate core–shell structures by the two-step emulsification process “emulsions of emulsions”. In the primary w/o emulsion, the aqueous phase represents the core to carry the antibiotic (imipenem and cilastatin). However, chloroform was used as a water immiscible organic phase, containing the shell-forming polymer (PCL or PLGA) and emulsion stabilizing agent (ethyl cellulose) [29]. The emulsifying agent (DMAB) was then added to the system to obtain the multiple-emulsion (w/o/w) and to achieve full diffusion of solvents. Subsequently, the solvent vaporization step was performed for nanoparticle solidification that occurred by solvent dispersion in association with polymer nanoprecipitation principles [23].

### Characterization of the prepared nanoparticles

**Fig. 1:**
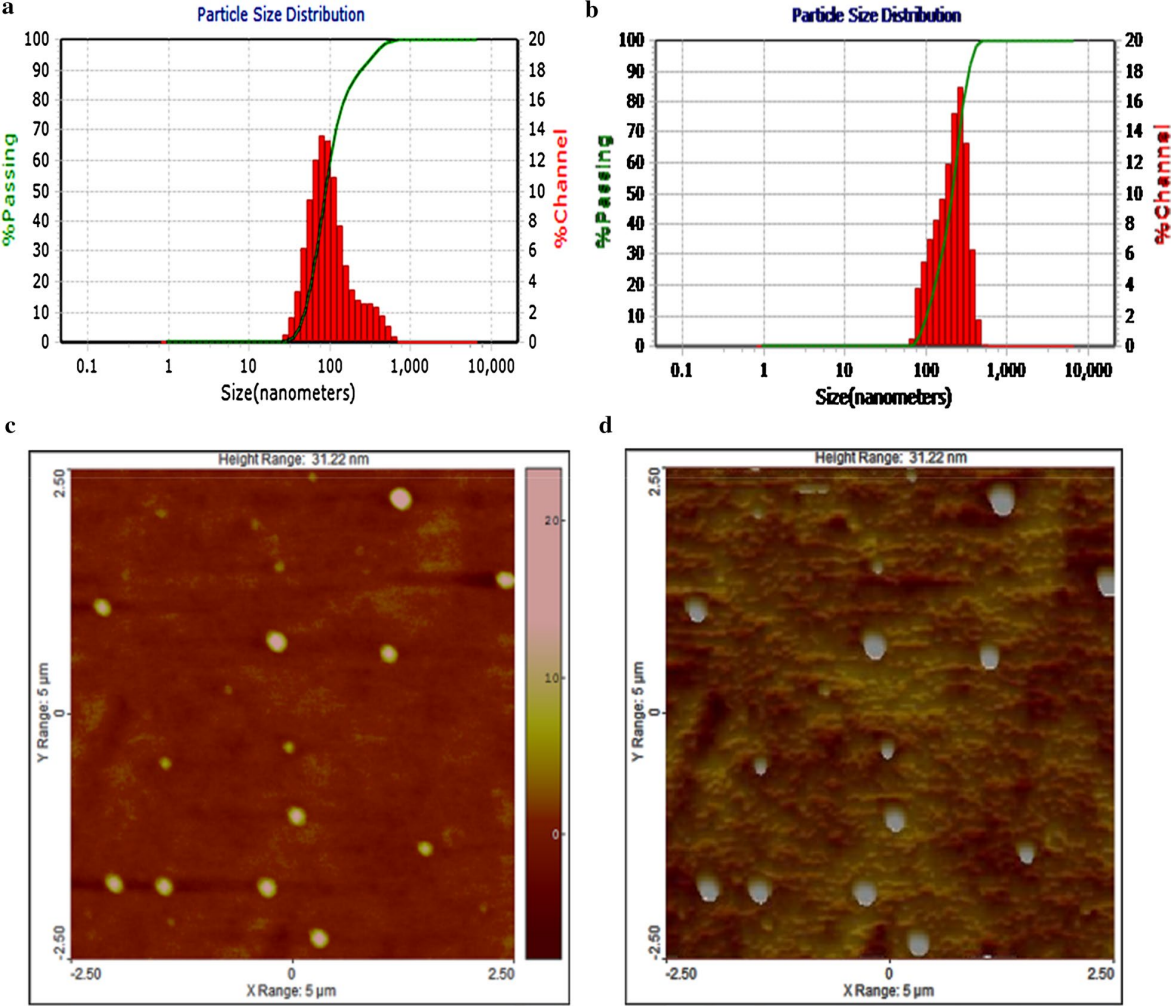
**a** Particle size distribution for imipenem/cilastatin loaded polycaprolactone nanoparticles as measured by laser diffraction particle analyzer. **b** Particle size distribution for imipenem/cilastatin loaded polylactide-co-glycolide nanoparticles as measured by laser differaction particle analyzer. **c** Image for imipenem/cilastatin loaded polycaprolactone nanoparticles as measured by atomic forced microscopy (AFM), a 2-D view and **d** the image in 3-D

The measured particle sizes and zeta potentials using laser diffraction particle analyzer are listed in Table 1. The prepared IMP/PCL nanoparticles showed smaller size and higher zeta potential compared to that of IMP/PLGA nanoparticles. However, both exhibited high encapsulation efficiency, drug loading and yield value. Meanwhile, Fig. 1 shows the atomic forced microscopy image for IMP/PCL nanoparticles. The contour photo views that the shape of PCL nanoparticles is well defined and have a distinctive spherical structure and a smooth surface. Furthermore, an acceptable match was observed in the mean diameter as measured by both laser diffraction particle sizer (Fig. 1a) and atomic forced microscopy (Fig. 1c).

**Table 1:** Physical characterization of imipenem/cilastatin loaded polycaprolactone (IMP/PCL), and imipenem/cilastatin loaded polylactide-*co*-glycolide (IMP/PLGA) nanoparticles

Used polymer	Size (nm)	Zeta potential (mv)	Encapsulation efficiency (EE) %		Drug loading (DL) %		Nanoparticle yield (NY) %
			Imipenem	Cilastatin	Imipenem	Cliastatin	
PCL	132 ± 20	17 ± 1.6	83 ± 2.2	81 ± 1.2	17.7 ± 0.6	17.3 ± 0.7	93 ± 2.7
PLGA	348 ± 65	15 ± 0.6	76 ±1.2	77 ± 3.8	17.2 ± 1.3	17.6 ± 0.9	88 ± 1.9

*PCL* polycaprolactone, *PLGA* polylactide-*co*-glycolide

**Fig. 2:**
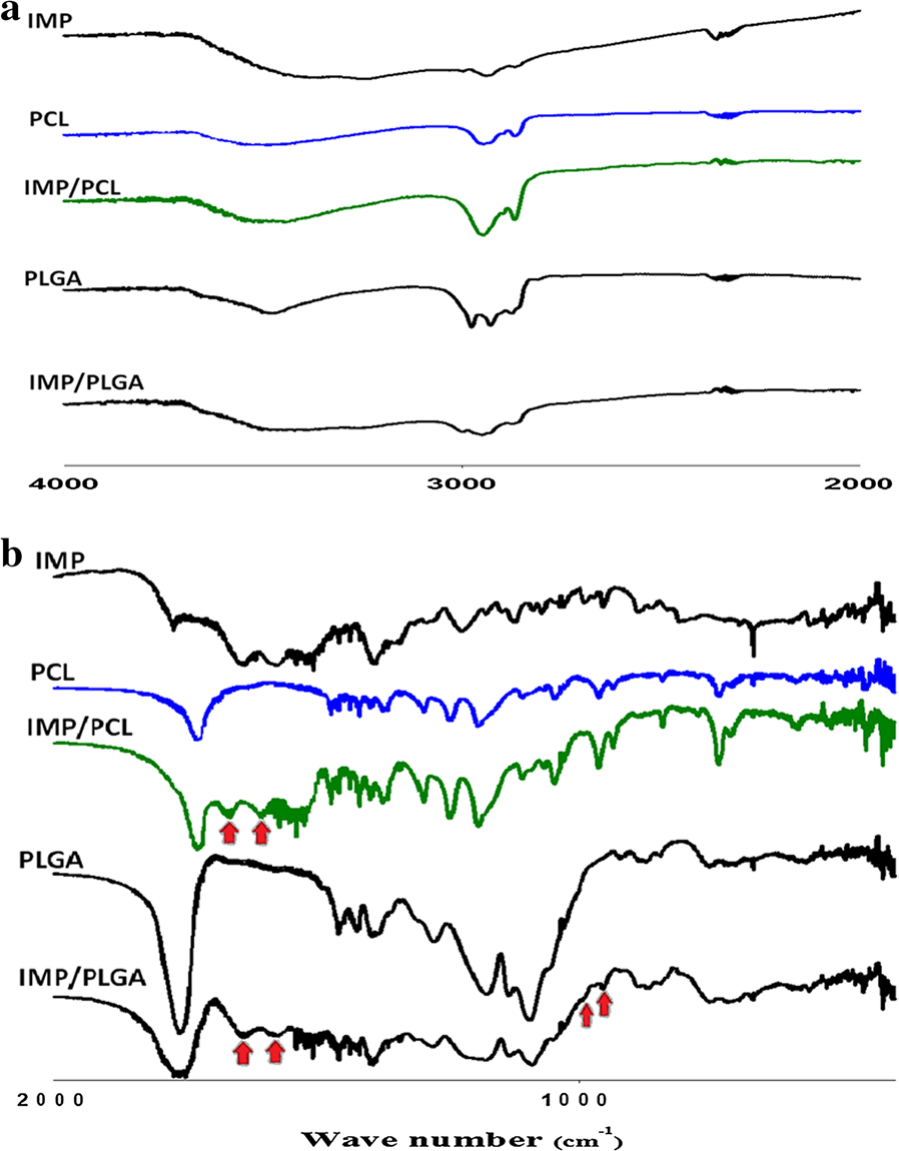
FTIR spectrum of imipenem/cilastatin (IMP), polycaprolactone nanoparticles (PCL), imipenem/cilastatin loaded polycaprolactone nanoparticles (IMP/PCL), polylactide-*co*-glycolide nanoparticles (PLGA), and imipenem/cilastatin loaded polylactide-*co*-glycolide nanoparticles (IMP/PLGA). **a** The spectrum from 4000 to 2000 cm^−1^. **b** The spectrum from 2000 to 400 cm^−1^

FTIR spectrum for IMP, PCL nanoparticles, IMP/PCL, PLGA nanoparticles and IMP/PLGA are shown in Fig. 2. As it observed the imipenem/cliastatin had characteristic peaks/bands (cm^−1^) that were attributed to vibration of OH stretching (3400 and 3250), N–H stretching (2950), C–H stretching (2860), C=O stretching (1776 and 1737), amide bands (1655 and 1580), C=N stretching (1460 and 1440), C=C stretching (1395 and 1230), N–C–S vibration (1070/1030), C=C bending (991 and 952), C–S stretching (892 and 809) C–N–H bending (727), C–S stretching (697) and C–O–H bending (666). The characteristic peaks/bands for PCL appear in both PCL nanoparticles and IMP/PCL nanoparticles which are OH stretching (3600–3200), C–H stretching (2950 and 2860), C=O stretching (1725), C–H symmetric/asymmetric deformation (1474, 1425, 1400 and 1370), C–O–C stretching (1297, 1246, 1194), C–O stretching (1108, 1070 and 1050), C–H bending (965 and 935), and C–C stretching (842 and 733). Likewise, the characteristic peaks/bands for PLGA appear with both PLGA nanoparticles and IMP/PLGA nanoparticles which are OH starching (3650–3250), C–H stretching (2980, 2930 and 2878), C=O stretching (1760), C–H symmetric/asymmetric deformation (1460 and 1425), C–O–C stretching (1395, 1275, 1175 and 1095), CH_3_ asymmetric rocking (1070/1030), C–O stretching (1054), and C–C stretching (872 and 751).

The encapsulation of IMP in the IMP/PCL nanoparticles was confirmed by the association of amide I band (1655) and amide II band (1580) in the spectrum. Also, the association of IMP in the IMP/PLGA nanoparticles was confirmed by the amide I band (1655), amide II band (1580) and the C=C bending (991 and 952). Assignment of the imipenem peaks in both IMP/PCL and IMP/PLGA nanoparticles confirms the encapsulation of imipenem and demonstrates the thermodynamic stability for the chemical structure of encapsulated imipenem [17].

**Fig. 3:**
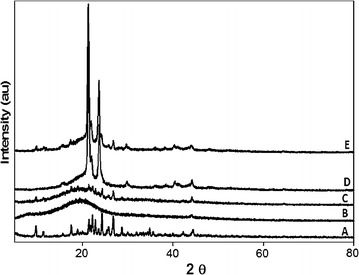
X-ray diffractograms for imipenem/cilastatin (*A*), polylactide-*co*-glycolide nanoparticles (*B*), imipenem/cilastatin loaded polylactide-*co*-glycolide nanoparticles (*C*), polycaprolactone nanoparticles (*D*) and imipenem/cilastatin loaded polycaprolactone nanoparticles (*E*)

X-ray diffractographs for IMP, PLGA, IMP/PLGA, PCL and IMP/PCL nanoparticles are shown in Fig. 3. The IMP showed its crystalline peaks at diffraction angles (2θ) of 9.6°, 11.3°, 17.5°, 18.9°, 21.3°, 22.2°, 22.8°, 23.5°, 24.3°, 25.7°, 26.7°, 28.6°, 34.8°, 42.1° and 44.4°. The prepared PLGA nanoparticles were amorphous and did not show any crystalline peaks, nonetheless, IMP/PLGA nanoparticles showed the IMP characteristic peaks but at low intensity. The PCL nanoparticles showed sharp and intense crystalline peaks at 21.4°, 21.9° and 23.6°, meantime, all these peaks in addition to IMP characteristic peaks were detected in IMP/PCL. The presence of IMP peaks within the pattern of both IMP/PLGA and IMP/PCL confirmed the crystalline pattern of the encapsulated IMP within the prepared nanoparticles and this is also in agreement with the previously published reports [17].

### Antibacterial activity

**Fig. 4:**
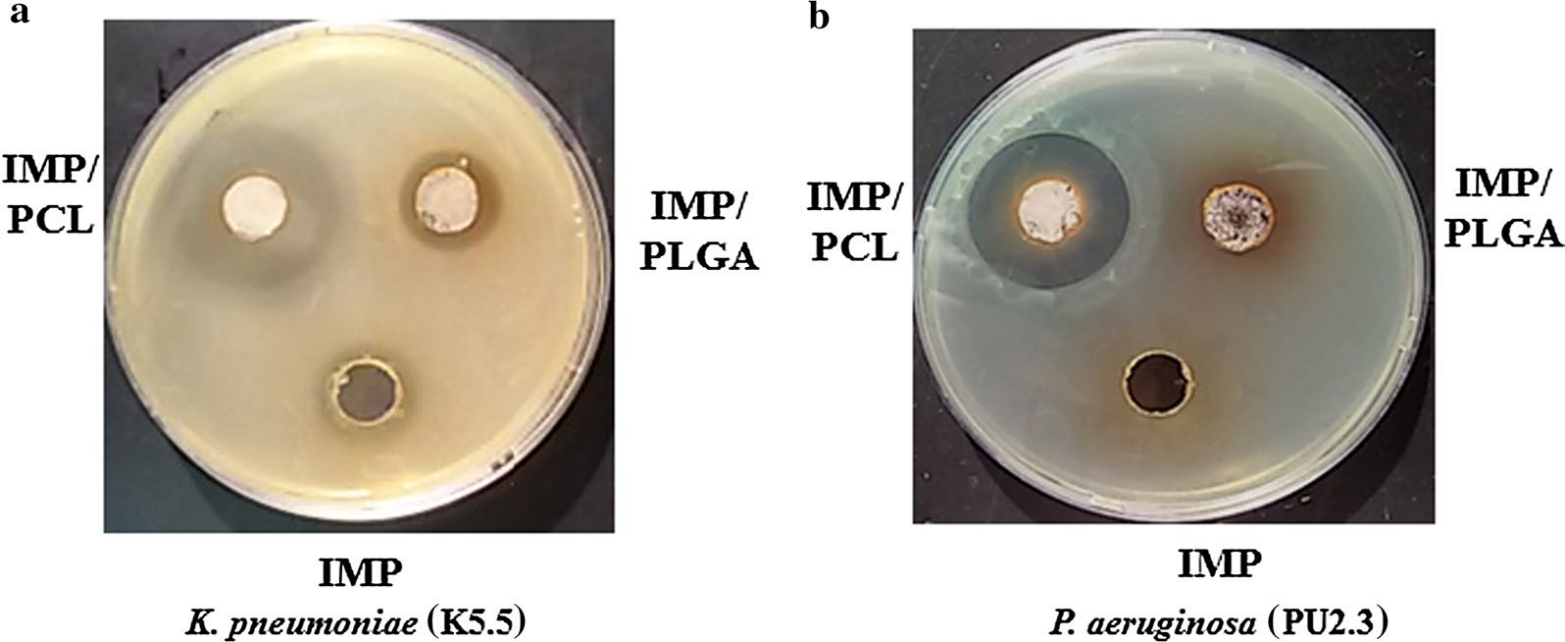
Antimicrobial screening of the formulated imipenem/cilastatin loaded polycaprolactone (IMP/PCL) and imipenem/cilastatin loaded polylactide-*co*-glycolide (IMP/PLGA) nanoparticles against *Klebsiella pneumoniae*, KMU5.5 (**a**) and *Pseudomonas aeruginosa* PUMU2.3 (**b**) compared to imipenem/cilastatin (IMP). IMP/PCL retained antimicrobial activity against both isolates

The initial screening for the prepared nanoparticles indicated that clinical isolates which were non-susceptible to IMP, but IMP/PCL showed marked antimicrobial activity against carbapenem-resistant isolates (Fig. 4). Table 2 displays the MIC values as measured through the broth micro-dilution technique, for IMP/PCL and IMP/PLGA compared to IMP. The obtained results illustrate that IMP inhibited the growth of PSMU8.0 isolate with MIC of 40 μg/ml, while, PWMU2.3 and PUMU2.3 isolated possessed high-level of resistance and showed MIC equals 625 μg/ml. In addition, IMP showed MIC value ranged from 80 to 625 μg/ml against the tested *Klebsiella pneumoniae* isolates. Meantime, IMP/PCL was more effective than IMP as the susceptibility of both *P. aeruginosa* and *K. pneumoniae* significantly increased (Table 2). The MIC of IMP/PCL has decreased by five to seven folds compared to the free IMP. IMP/PLGA formulation was less effective than IMP/PCL and was only able to decrease the MIC of KMU4.5 (two folds) as well as PWMU2.3 and PSMU80 (1-2 folds) from all of the tested isolates (Table 2). At the same time, the control nanoparticles either PLGA or PCL revealed no activity on the bacterial growth, highlighting that the antimicrobial effects were only obtained from the encapsulated drug itself.

**Table 2:** The minimal inhibitory concentrations (MIC) of imipenem/cilastatin loaded polycaprolactone (IMP/PCL), and imipenem/cilastatin loaded polylactide-*co*-glycolide (IMP/PLGA) compared to imipenem/(IMP) against *Klebsiella pneumoniae* and *Pseudomonas aeruginosa* isolates

	MIC (µg/ml)		
	IMP/PCL	IMP/PLGA	IMP
*Klebsiella pneumoniae*			
ATCC4352	0.6	1.25	0.6
KMU5.5	5	312.5	312.5
KMU4.5	2.5	156.5	625
KMU2.3	2.5	80	80
*Pseudomonas aeruginosa*			
PAO1	1.25	1.25	1.25
PWMU2.3	20	312.5	625
PUMU2.3	10	625	625
PSMU8.0	1.25	20	40

These results are also in accordance with reported outcomes that have been published previously. Xiong et al. had been demonstrated that a vancomycin-loaded PCL nanoparticles are more effective in penetrating cells infected with *Staphylococcus aureus* compared to free vancomycin [30]. Also, acrylated penicillin G polymer exhibits potent antimicrobial activity against MRSA [16]. Nano-penicillin with particle size 70 nm exhibits higher bactericidal activity than the bulk penicillin [31]. As it has been reported, the ability of imipenem to pass through the Gram-negative bacterial membrane is mainly attributed to its low molecular weight and zwitterion nature [32]. Imipenem molecules penetrate the bacteria through membrane transporting proteins (porins) which act as pores/channels for selective/nonselective diffusion of molecules as nutrients, such as outer membrane porins (OMPs) [24, 33]. Some of these porins are nonspecific such as omp F and omp C whereas others are special for certain molecules such as OprD which is specifically expressed in *P. aeruginosa* [6]. Concurrently, it was revealed that gained bacterial resistance to imipenem is associated with alteration in the structure of the imipenem binding and penetration part in porins [6, 34] such a mutational change resulting in specific bacterial identification to imipenem molecules with the loss of imipenem transmembrane entrance ability [34]. Nonetheless, encapsulated imipenem will not be easily identified by bacteria and is able to disguisedly diffuse through the membrane porins. Consequently, the observed enhancement in the antibacterial action of IMP/PCL could be associated with the enhanced membrane permeability associated with nanosize-encapsulated imipenem [14, 15, 30]. Meantime, the bacterial porins act as size exclusion filters and the size of the prepared particles plays that role in penetrability through the membrane, therefore, IMP/PCL nanoparticles (132 ± 20 nm) were more penetrable than that of the IMP/PLGA (348 ± 65 nm).

### Protection from carbapenemases

**Fig. 5:**
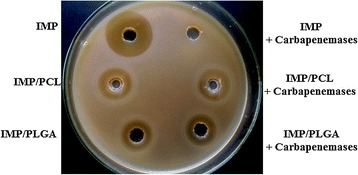
Effect of carbapenemases on activity of imipenem/cilastatin (IMP), imipenem/cilastatin loaded polycaprolactone nanoparticles (IMP/PCL) and imipenem/cilastatin loaded polylactide-*co*-glycolide nanoparticles (IMP/PLGA) against *Escherichia coli* ATCC 25922

Production of carbapenemase is also included as an essential factor in carbapenem-resistance for Gram-negative bacteria [7, 35]. Incorporation of IMP into the nanocapsules could provide protection from bacterial degrading enzymes. In order to address this hypothesis, IMP and IMP formulations were screened against *E. coli* sensitive isolate in the presence and the absence of carbapenem degrading enzymes (Fig. 5). The image shows that IMP, IMP/PCL and IMP/PLGA, had the same antimicrobial effect against the carbapenem-susceptible *E. coli*. When they were treated with the hydrolyzing enzymes, IMP lost all its activity while both nanoparticles retained their inhibitory effects. This could be attributed to the protective capacity of the polymeric nanoparticle to the incorporated antimicrobial agent from the inactivation by hydrolyzing enzymes without affecting its antibacterial properties. As such loaded imipenem molecules remained effective against resistant Gram-negative isolates. Likewise, coating penicillin G in polyacrylate nanoparticles provides protection to the loaded antibiotic from extracellular penicillinases and retains penicillin efficacy [16].

### Mutation inhibition effect

Above the minimal inhibitory concentration, bacteria exhibit a selective emergence of resistant sub populations. The mutation prevention concentration represents the concentration that suppresses the emergence of resistance mutations and equivalent to the MIC of the strains. On that ground, we screened the ability of IMP/PCL to induce antibiotic mutation in comparison to IMP control. IMP induced the resistance in *K. pneumoniae* isolate KMU2.3 and enhanced the phenotypic switching to high-level antibiotic resistance rapidly and irreversibly. The MPC of KMU2.3 emerging mutants was 320 µg/ml with twofolds increase in the MIC of IMP. However, IMP/PCL did not induce mutation and the MPC was equal to the MIC (2.5 µg/ml) in KMU2.3.

Previous studies revealed the ability of imipenem and meropenem to induce mutational changes in Gram-negative bacilli [24, 33]. This mutational change has been attributed to various resistance mechanisms such as changing cell permeability [36], induction of carbapenemase [37] or expression of metallic β-lactamases from an integron [35]. However incorporation of imipenem into polymeric nanoparticle was able to prevent such a mutational changes.

### Time kill assay

**Fig. 6:**
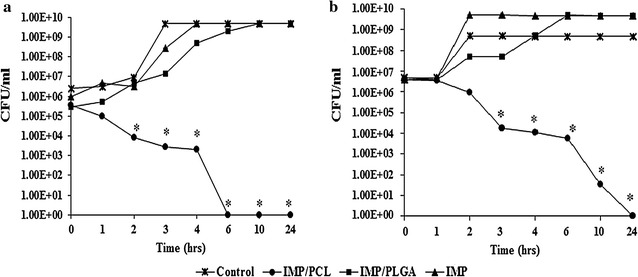
Time kill assay of imipenem/cilastatin loaded polycaprolactone nanoparticles (IMP/PCL), and imipenem/cilastatin loaded polylactide-*co*-glycolide nanoparticles (IMP/PLGA) against **a** *Klebsiella pneumoniae* KMU5.5 and **b** *Pseudomonas aeruginosa* PUMU2.3. Low concentration of IMP/PCL (5-10 µg/ml) caused rapid and significant decrease in bacterial count within 2–3 h of interaction (*significant difference *P* < 0.05)

The rate of microbial killing following antibiotic treatment is so critical for avoiding any future resistance from bacteria. For that reason, we tracked bacterial viability in the presence of IMP, IMP/PLGA and IMP/PCL compared to control untreated cultures. As shown in Fig. 6, treating *K. pneumoniae* KMU5.5 isolate with IMP/PCL caused significant decline (P < 0.01) in the bacterial growth within 2 h of incubation and complete bacterial killing after 6 h. However, IMP and IMP/PLGA did not affect bacterial growth (Fig. 6a). Similarly, the count of *Pseudomonas aeruginosa* PUMU2.3 significantly decreased after 3 h of incubation with IMP/PCL (P < 0.01), within 6 h the count of PUMU2.3 was reduced from 2 × 10^8^ to 5.5 × 10^3^ CFU/ml and declined to 45 colonies by 10 h. Coating of IMP with PCL increased its killing capacity. Instant microbial killing induced by IMP/PCL could be attributed to bacterial hydrophobic affinity for the PCL polymer and the nano-size/charge of the formulated imipenem, which assist the rapid penetration through the bacterial outer membrane [5].

### Anti-adhesion activity

**Fig. 7:**
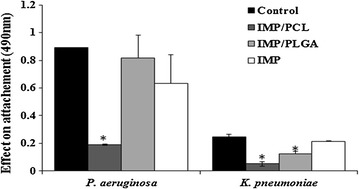
Effect of imipenem/cilastatin (IMP), imipenem/cilastatin loaded polycaprolactone nanoparticles (IMP/PCL), and imipenem/cilastatin loaded polylactide-*co*-glycolide nanoparticles (IMP/PLGA) on biofilm formation. IMP/PCL significantly eliminated the adhesion of *Pseudomonas aeruginosa* and *Klebsiella pneumoniae* and inhibited biofilm assembly (*significant difference P < 0.05)

Biofilm is a disseminated microbial growth that is difficult to penetrate and develops resistance to conventional treatment. Various strategies have been investigated to enhance the antimicrobial to microbial biofilms especially those biofilms formed on the implanted medical devices [38]. As demonstrated in Fig. 7, the influence of IMP and nano-formulated imipenem at sub-inhibitory concentrations (1/4 MIC) on the attachment of *K. pneumoniae* and *P. aeruginosa* were evaluated. IMP non-functionalized antibiotic maintained bacterial ability for biofilm synthesis. The adhesion of KMU5.5 and PUMU2.3 in the presence of IMP/PCL was significantly (P < 0.01) reduced by 74 and 78.4%, respectively, compared to the control untreated cultures.

Formulation of nano-therapy increases the solubility and decrease the aggregation of the antimicrobial agents hence, improve their penetration and efficacy. Nano-formulation of IMP resulted in reducing the particle size of imipenem and enhanced the penetration of the loaded antibiotic [9]. In addition to the hydrophobic nature of PCL chains that assists the rapid penetration of the antibiotic and rupture of the bacterial cell wall so prevents microbial colonization and block biofilm formation [13].

### In vitro drug release

**Fig. 8:**
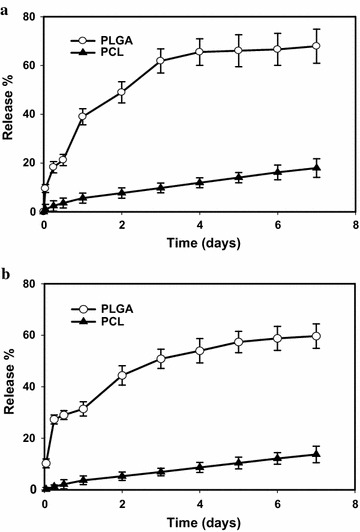
Cumulative percent of released imipenem (**a**) and cilastatin (**b**) from the prepared polymeric nanoparticles polycaprolactone (PCL) and polylactide-*co*-glycolide (PLGA) in phosphate buffer saline (pH = 7.4) as a release media at 37 °C. *Error bars* represent the standard deviation of the mean of measurements from four samples

Diffusion-driven release profiles of both imipenem and cilastatin from the prepared nanoparticles are demonstrated in Fig. 8. With PLGA nanoparticles, fast release occurs within the initial phase (the first three days) followed by a much slower release period. The release profiles show that 61.8 and 50.8% of the loaded imipenem and cilastatin, respectively, released within the first 3 days and 67.8 and 59.6%, released at the day 7. Meanwhile, PCL nanoparticles demonstrated a slow diffusion rate all over the release study periods. The release profiles show that only 9.7 and 6.9% of the loaded imipenem and cilastatin, respectively, released within the first 3 days and 17.9 and 13.7% of the loaded imipenem and cilastatin, respectively, released at the day 7. This turns out that PCL nanoparticles are very good at slowly releasing imipenem/cilastatin over extended periods of time.

PLGA is relatively amorphous low molecular weight polymer with reasonable hydrophilicity [39], whereas, PCL is a semi-crystalline high molecular weight polymer with reasonable hydrophobic nature [40]. Hence, hydrophilic drug (imipenem/cilastatin) was incorporated within the polymeric shell of PLGA nanoparticles (poor entrapment) or in the core of PCL nanoparticles (good entrapment). Shell and surface associated drug is adjacent to the PBS and is easily released, depending on water solubility and water imbibition within the nanoparticles shell (polymeric layer) [41]. Therefore, the initial burst release of imipenem from PLGA is mainly attributed to the diffusion from the outer most layers of the nanoparticles as well as emancipation on the external layers of the nanoparticles. In addition to the PLGA bulk degradation that is associated with a gradual decline of its molecular weight; even without weight loss or soluble monomer formation [39]. Such a fast release phase is associated with a linear slow release phase, where the drug diffusion only was occurring. On the other hand, the slow release rate from PCL nanoparticles can be attributed to the hydrophobic properties of drug depleted shell layers [40].

## Conclusion

Imipenem/cilastatin encapsulated polylactide-*co*-glycolide and polycaprolactone nanoparticles were successfully prepared by double emulsion evaporation method. Antibacterial efficacy evaluation results showed that polycaprolactone nanoparticles were more effective than polylactide-*co*-glycolide nanoparticles and free drug, against all of the tested resistant isolates. Hence, the antimicrobial efficacy of carbapenems can be regained to smash the developing resistant isolates if the polymeric nanoencapsulation of the drug is virtually adopted. More effective therapies/clinical outcomes are expected from the strategy of using carbapenem-encapsulated polycaprolactone nanoparticles in comparison with using the current marketed formule administered in the hospitals. It cannot be overlooked that such a suggested application still requires further strong and dependable in vivo testing of antimicrobial activity, for confirming the efficacy and the validity for pharmaceutical production. A highlighted area for future perspective is applying carbapenem-loaded polycaprolactone nanoparticles in vivo in different local/systemic infections using suitable animal models and our study in this regard is still undergoing.
